# Diencephalic integrity explains aspects of hippocampal amnesia

**DOI:** 10.1093/cercor/bhag069

**Published:** 2026-06-19

**Authors:** Georgios P D Argyropoulos, John P Aggleton, Christopher R Butler

**Affiliations:** Memory Research Group, Nuffield Department of Clinical Neurosciences, University of Oxford, John Radcliffe Hospital, Headington, OX3 9DU, Oxford, United Kingdom; School of Psychology, Faculty of Natural Sciences, University of Stirling, Cottrell Building, Stirling, FK9 4LA, Scotland, United Kingdom; School of Psychology, Cardiff University, 70 Park Pl, Cardiff, CF10 3AT, United Kingdom; Memory Research Group, Nuffield Department of Clinical Neurosciences, University of Oxford, John Radcliffe Hospital, Headington, OX3 9DU, Oxford, United Kingdom; The George Institute for Global Health, Imperial College London, 58 Wood Ln, London W12 7RZ, United Kingdom; Department of Brain Sciences, Imperial College London, Du Cane Rd, London W12 0NN, United Kingdom; Departamento de Neurología, Pontificia Universidad Católica de Chile, Marcoleta 367, 8320165 Santiago, Región Metropolitana, Chile

**Keywords:** amnesia, hippocampus, mammillary bodies, thalamus, white matter

## Abstract

Research on amnesia has been fundamental in establishing the role of the human hippocampus in memory. Even though other structures within the hippocampal-diencephalic-cingulate network also play a role in episodic memory, studies of hippocampal amnesia often ignore the importance of changes in this broader network. In a large cohort of patients (*n* = 38) with hippocampal damage due to autoimmune limbic encephalitis, we previously found that amnesia was predominantly explained by resting-state functional abnormalities across this network. Here, we examined the integrity of individual diencephalic nuclei and white matter pathways, and its relationship with memory function. We found atrophy in the mammillary bodies, and the anterior, laterodorsal, pulvinar, and dorsomedial thalamic nuclei. Atrophy was often as pronounced as that in the hippocampal formation. Diencephalic volumes predicted memory over and above any hippocampal/subicular subfield volume estimate. White matter was compromised within and beyond this network. Fornix integrity was linked to diencephalic and hippocampal volumes, but not to recollection/recall. We strongly advise caution in employing the term “focal hippocampal damage” in cognitive neuroscience, and highlight the need to study the significance of plausibly knock-on effects in specific diencephalic nuclei and white matter tracts within broader circuits.

## Introduction

Hippocampal amnesia has taken center stage in memory neuroscience since patient H.M (Scoville and Milner 1957), and is commonly explained on the basis of hippocampal volume reduction (Wixted and Squire 2004; Gold et al. 2006; Patai et al. 2015; Dede et al. 2016b; Miller et al. 2017). However, recent work (echoing earlier insight: Warrington and Weiskrantz 1982; Heilman et al. 1990; Clarke et al. 1994) emphasizes functional dysconnectivity between the affected hippocampal formation and the rest of the brain as explaining hippocampal amnesia (Hayes et al. 2012; Rudebeck et al. 2013; Henson et al. 2016; Miller et al. 2020). Moreover, there is evidence for abnormalities beyond the medial temporal lobe that may follow hippocampal damage (Cormack et al. 2005; Rosenberg et al. 2006; Mueller et al. 2010; Morgan et al. 2015; Romeo et al. 2019; Tung et al. 2022). Extra-hippocampal damage within the “extended hippocampal system” (Aggleton and Brown 1999; “hippocampal-diencephalic-cingulate network”: Bubb et al. 2017; “revised Papez circuit”: Aggleton et al. 2022), may also impair episodic memory (Aggleton and Brown 1999; Tsivilis et al. 2008; Kafkas et al. 2020): fornical damage may impair recollection (retrieval of contextual details of events) and thus recall (Carlesimo et al. 2007; Tsivilis et al. 2008; Vann et al. 2009), but not familiarity (the sense of having encountered a stimulus, without retrieving contextual details) and recognition (supported by recollection and familiarity).

Despite acknowledging the need to examine this network across memory-impaired patients (Jonin et al. 2018; Basile et al. 2020), there remains little research on the explanatory potential of such knock-on effects in hippocampal amnesia (Argyropoulos et al. 2019; Harms et al. 2023). These effects may occur via Wallerian degeneration and/or seizure activity (Gross 2011; Elliott et al. 2018; Benear et al. 2020). Such research may help resolve debates on hippocampal contributions to processing remote memories (Squire and Bayley 2007; Dede et al. 2016a; Moscovitch et al. 2016), working memory (Squire 2017; Yonelinas et al. 2024), or to material- (Lee et al. 2006; Bird and Burgess 2008; Lee et al. 2012; Mullally et al. 2012; Maguire and Mullally 2013; Kim et al. 2015; Urgolites et al. 2017), and/or process-specific roles of the hippocampal-diencephalic-cingulate network (Lee et al. 2006; Kopelman et al. 2007; Wixted and Squire 2011; Patai et al. 2015; Kafkas et al. 2017; Argyropoulos and Butler 2020). It may also clarify similarities and discrepancies in the neuropsychological profile of hippocampal vs. extra-hippocampal damage, and how the interactions of those areas support memory (Winocur 1985; Aggleton and Brown 1999; Gold and Squire 2006; Kopelman et al. 2007; Aggleton et al. 2008).

In Argyropoulos et al. (2019), we had reported a large cohort (*n* = 38) characterized by hippocampal atrophy (which, beyond the volume reduction in the right entorhinal cortex, was focal within the medial temporal lobe) and residual amnesia due to autoimmune limbic encephalitis (aLE)—a disease studied as a “human model” of hippocampal damage (Maguire et al. 2006; Hassabis et al. 2007; Henson et al. 2016; Maguire et al. 2016; McCormick et al. 2016; Henson et al. 2017; McCormick et al. 2018; Lad et al. 2019; Spanò et al. 2020b) due to its focality (post-mortem and animal-model studies: Dunstan and Winer 2006; Park et al. 2007; Khan et al. 2009; Tröscher et al. 2017) relative to ischemia/anoxia or other encephalitides (Damasio and Van Hoesen 1985; Gitelman et al. 2001; Raschilas et al. 2002; Huang and Castillo 2008; Heinz and Rollnik 2015; Kelly et al. 2024). Acute clinical scans had disclosed very few abnormalities beyond the medial temporal lobes, and none in the diencephalon, unlike the significant diencephalic injury in other conditions causing hippocampal damage, such as early hypoxic-ischemic encephalopathy (Dzieciol et al. 2017; Meys et al. 2022). Post-acutely, however, we recorded whole-thalamic volume reduction and resting-state functional abnormalities, involving the cingulate. Surprisingly, it was these abnormalities, and not hippocampal volumes, that mostly explained amnesia.

Since Argyropoulos et al. (2019), methods designed to overcome the difficulties posed by conventional MRI protocols in automatically segmenting individual diencephalic nuclei have become available (Billot et al. 2020; Greve et al. 2021; Tregidgo et al. 2023; Vidal et al. 2024). In this study, we predicted that the key components of the revised Papez circuit with strong support for involvement in episodic memory (principal anterior thalamic nuclei, mammillary bodies, fornix; Aggleton et al. 2022), would show structural abnormalities, that their integrity would be associated with that of the hippocampal formation, and that these abnormalities would explain episodic memory impairment across patients. In addition to hippocampal volumes derived from gold-standard manual delineation in this cohort (Argyropoulos et al. 2019), we also estimated hippocampal/subicular subfield volumes, and whether they would show relationships with memory scores over and above diencephalic volumes. We also sought evidence consistent with a causal account, whereby hippocampal damage would compromise fornical integrity, which would, in turn, impact the mammillary bodies. Finally, we expected that abnormalities would be present even in anti-leucine-rich glioma inactivated (LGI1)-aLE, i.e. the type that is often the focus of many studies of hippocampal damage (Lad et al. 2019; Miller et al. 2020; Spanò et al. 2020a; Spanò et al. 2020b), as it may present with damage and symptoms more focally and homogeneously compared with other types (van Sonderen et al. 2016; Bastiaansen et al. 2017; Finke et al. 2017; van Sonderen et al. 2017).

## Materials and methods

### Participants

Our patient cohort (*n* = 38; 26M:12F; age at imaging: mean = 61.39; SD = 13.91 yr), presented in Argyropoulos et al. (2019, 2020), comprised individuals who had been diagnosed with aLE according to established criteria (Graus et al. 2016; S-Methods 1).

### MRI data acquisition

#### Structural MRI

All patients underwent structural brain imaging, along with 67 healthy controls (40M:27F; age at imaging: mean = 61.23; SD = 13.57 yr; controls vs. patients: M:F ratio: χ^2^ = 0.79, *P* = 0.374; age: t(103) = −0.06, *P* = 0.954). T1-weighted images were acquired using a Magnetization Prepared Rapid Gradient Echo sequence with a Siemens 3T Trio system and a 32-channel head coil (University of Oxford Centre for Clinical Magnetic Resonance Research; echo time = 4.7 ms, repetition time = 2,040 ms, 8° flip angle, field of view = 192 mm, voxel size = 1 × 1 × 1 mm). All patients and 35/67 controls were recruited by the Memory and Amnesia Project (University of Oxford). An additional 32 structural MRI datasets of controls were made available through the Oxford Project To Investigate Memory and Aging. Although acquisition protocols did not differ (Zamboni et al. 2013), our volumetric comparisons between all controls and patients include scan source as a covariate.

#### Diffusion MRI

All diffusion data were acquired under our Memory and Amnesia Project, involving 35/35 controls (age: mean = 55.14; SD = 14.04 yr; 25M:10F), and 37/38 patients (age: mean = 61.14; SD = 14.01 yr; 26M:11F; controls vs. patients: M:F ratio: χ^2^ = 0.01, *P* = 0.914; age: t(70) = 1.81, *P* = 0.074; age and sex were included in between-groups comparisons). We used a single-shot echo planar imaging sequence (64 slices; slice thickness = 2 mm, 0 gap; axially acquired; 64 directions at b = 1,500 s/mm^2^, repetition time = 8,900 ms; echo time = 94.8 ms; voxel size = 2 × 2 × 2 mm; field of view = 192 × 192 mm). One no-diffusion-weighted image at b = 0 s/mm^2^ was also acquired.

### Experimental design and statistical analysis

#### Automated diencephalic volumetry

We first compared controls and patients (ANCOVAs—covariates: age, sex, FreeSurfer’s estimated total intracranial volume [eTIV], scan source) on the volumes of the (left+right) mammillary bodies segmented with FreeSurfer-ScLimbic (Greve et al. 2021), and the thalamic segmentations estimated by the convolutional neural network tool developed in FreeSurfer (Tregidgo et al. 2023) (henceforth, “FreeSurfer-CNN”). FreeSurfer-ScLimbic is a deep-learning tool implemented in FreeSurfer (“mri_sclimbic_seg”; https://surfer.nmr.mgh.harvard.edu/fswiki/ScLimbic) that automatically segments several subcortical limbic structures using T1-weighted images (Greve et al. 2021) (available for 105/105 participants). FreeSurfer-CNN (https://surfer.nmr.mgh.harvard.edu/fswiki/ThalamicNucleiDTI) employs diffusion and T1-weighted MRI data (available for 37 patients and 35 controls). Using FreeSurfer 7.4.1, we first produced a bias-corrected, whole-brain structural T_1_-weighted MRI image (“norm.mgz”), a whole-brain segmentation (“aseg.mgz”) using “recon-all”, and a T_1_-based FreeSurfer segmentation (Iglesias et al. 2018), using “segmentThalamicNuclei.sh” (https://freesurfer.net/fswiki/ThalamicNuclei). We then used “TRActs Constrained by UnderLying Anatomy” (Yendiki et al. 2011; Maffei et al. 2021) to derive a fractional anisotropy volume (“dtifit_FA.nii.gz”) and a 4D volume containing the principal direction vector for each DTI voxel (“dtifit_V1.nii.gz”). The segmented nuclei comprise the central lateral, central medial, centromedian, lateral geniculate, lateral posterior, laterodorsal, limitans (suprageniculate), medial geniculate, mediodorsal lateral (parvocellular), mediodorsal medial (magnocellular), parafascicular, pulvinar anterior, pulvinar inferior, pulvinar lateral, pulvinar medial (medial segment—henceforth, “m”; lateral segment—henceforth, “l”), reuniens (medial ventral), ventral anterior, ventral anterior magnocellular, ventral lateral anterior, ventral lateral posterior, ventral posterolateral nuclei, along with an “anteroventral” segmentation, which, however, includes the anterior medial and anterior dorsal nuclei (Iglesias et al. 2018)—we will thus refer to this segmentation as the “principal anterior nuclei” instead. We applied the Holm–Bonferroni sequential correction (Holm 1979) for the number of tests (*n* = 24) conducted (henceforth, “p-corr(24)”). We also examined whether these differences could be conceptually replicated using HIPS-THOMAS (Vidal et al. 2024) for the thalamus, and HypothalamicSubunits for hypothalamic segmentations (Billot et al. 2020; S-Methods 2). Comparing these segmentation methods is beyond the scope of this paper (Argyropoulos et al. 2025).

#### WM integrity

A standard preprocessing pipeline for single-shell single-tissue constrained spherical deconvolution was followed in MRtrix3 (Tournier et al. 2019) (S-Methods 3). Connectivity-based fixel enhancement (a threshold-free cluster-enhancement-like approach) (Smith and Nichols 2009) was involved, which used probabilistic tractography to identify structurally connected fixels that share underlying anatomy. FWE-corrected *P*-values (<0.05) were then assigned to each fixel using non-parametric permutation testing (5,000 permutations). As in recent studies (Zarkali et al. 2020; Andica et al. 2021), we selected fiber density and cross-section (FDC) as the measure of interest, since it reflects both microstructural and macrostructural changes, representing a tract’s overall ability to relay information (Raffelt et al. 2017). To conceptually replicate the findings of our fixel-based analysis, we employed another two whole-brain automated analyses of WM integrity (voxel-based morphometry, tract-based spatial statistics), along with deterministic tractography to manually reconstruct key tracts of interest (S-Methods 4–6).

#### Structure–structure/behavior relationships

We examined the relationship of impaired episodic memory with reduced diencephalic volumes (n composite memory scores = 5; n reduced diencephalic volumes = 8). Volumes were residualized against the same regressors as those included in the ANCOVAs above. Correlational analyses (conducted separately for patients) employed our two composite scores—“anterograde retrieval” and “remote autobiographical memory”, which were shown to be better explained by extra-hippocampal abnormalities in our cohort (p-corr(16)). The former was the average of standardized age-scaled scores on 13 (sub)tests, on all of which the patient cohort showed impairment (Argyropoulos et al. 2019): (i) visual recall: Doors and People—Shapes (Baddeley et al. 1994), Rey–Osterrieth Complex Figure Copying Test (Rey 1959) Immediate and Delayed Recall; (ii) visual recognition: Doors and People—Doors (Baddeley et al. 1994), Warrington Topographical Memory test (Warrington 1996); (iii) verbal recall: Wechsler Memory Scale III—Logical Memory I/II, Word List I/II (Wechsler 1997), Doors and People—People (Baddeley et al. 1994); (iv) verbal recognition: Doors and People—Names (Baddeley et al. 1994), Wechsler Memory Scale III—Word List II Recognition (Wechsler 1997), Warrington Recognition Memory Tests—words (Warrington 1984). The latter was the sum of autobiographical memory scores for Childhood and Early Adulthood (Kopelman et al. 1989; see Argyropoulos et al. (2019) for the reasons why the “Recent” phase scores were disregarded). These correlations were then iterated, after decomposing the anterograde composite score into verbal/visual recall/recognition (p-corr(40)) and also by examining laterality-specific relationships (p-corr(80)).

We further estimated hippocampal/subicular subfield volumes, in order to ascertain whether these, unlike our gold-standard manually delineated hippocampal volumes (Argyropoulos et al. 2019), strongly predicted episodic memory scores (a fortiori, over and above the diencephalic volumes here). We did so in a deliberately exploratory manner, not correcting for multiple comparisons, while recognizing that 1mm^3^-resolution MRIs may be too low-quality for automated subfield volume estimates (Wisse et al. 2021). We first used FreeSurfer’s “Subregions Segmentation” (Iglesias et al. 2015) to derive subfield segmentations of the hippocampal formation. This tool (“segment_subregions”; https://surfer.nmr.mgh.harvard.edu/fswiki/SubregionSegmentation) was used for segmentation of MR images pre-processed through the FreeSurfer “recon-all” pipeline. It outputs segmentations for the right/left hippocampal formation: amygdala transition area, CA1/CA3/CA4 head/body, subiculum head/body, presubiculum head/body, molecular layer head/body, granule cell and molecular layer of the dentate gyrus head/body, hippocampal fissure, parasubiculum, tail, and fimbria. We employed this tool, given the widespread use and documentation of FreeSurfer pipelines, and the fact that approximately 95% of subfield segmentations have been found to have “excellent” numerical reliability (Kahhale et al. 2023). We excluded the fimbria (WM) from our volumetric comparisons. A series of ANCOVAs (covariates: age, sex, scan source, and eTIV) on raw total (left+right) volumes showed that all subfields, apart from the parasubiculum and the hippocampal fissure, were atrophic (15%–26%; p-corr(18) < 0.05; *η^2^_p_* = 0.11–0.36; Table S1). We then used the reduced (left+right) subfield volumes (residualized against age, sex, scan source, and eTIV) that showed the (numerically) highest correlation coefficient with a composite memory score of interest (Fig. S1) as a control variable in partial correlation analyses of the relationships between diencephalic volumes and memory scores. We also conducted a series of mediation analyses, to investigate whether any relationships observed between these hippocampal/subicular subfield volumes (predictor) and episodic memory scores (outcome) were mediated by the affected diencephalic volumes across patients. Mediation analyses rely on the assumption of causality between the predictor and the mediator (Judd and Kenny 1981; Baron and Kenny 1986; MacKinnon et al. 2007). A default number of 5,000 bootstrap samples was used to calculate the standard errors and confidence intervals.

For the purposes of examining structure–structure/behavior relationships, we used a tract-of-interest analysis. Major tracts (*n* = 70/72 totally generated, excluding the inferior cerebellar peduncle, as inferior cerebellar slices were clipped from the field of view) were automatically delineated on the WM fiber orientation distribution template using TractSeg (Wasserthal et al. 2018). Delineated fiber tracts were then converted into fixel masks after using the “tck2fixel” command in MRtrix3 (Tournier et al. 2019), and subsequently thresholding this fixel image to create a binary tract fixel mask. We extracted mean tract FDCs, and, for the purposes of reducing the number of tracts and hence the number of tests conducted, we collapsed across hemispheres, and also derived a single mean FDC for the corpus callosum from the 7 available (“CC 1–7”). We then compared controls and patients in a series of ANCOVAs (covariates: age, sex; p-corr(33)) to identify the tracts affected in aLE. We then entered the mean FDC (residualized for age and sex) of the affected tracts (*n* = 25) into correlational analyses with the volumes (residualized for age, sex, scan source, and eTIV) of the mammillary body and the hippocampal formation (p-corr(50)), to identify tracts whose integrity was associated with that of the mammillary body and the hippocampal formation. We predicted that the integrity of the fornix would show strong relationships with that of both the hippocampus and the mammillary bodies across patients, and that the effects of hippocampal volume (predictor) on mammillary body volume (outcome) would be mediated by the FDC of the fornix, given our hypothesis that any reduction in the volume of mammillary bodies would follow as a knock-on effect of damage in the hippocampal formation and subsequently in the fornix. A default number of 5,000 bootstrap samples was used to calculate the standard errors and confidence intervals.

We ran a series of correlations between the mean FDC (residualized) of the affected tracts (*n* = 25) and anterograde retrieval/remote autobiographical memory (p-corr(50)). In a post hoc, exploratory fashion, we examined whether the integrity of the fornix also predicted Recollection and/or Familiarity for Faces, Scenes, and/or Words across the 8 aLE patients who had completed custom-made recognition memory tasks, reported in Argyropoulos et al. (2022). Mean FDC (residualized) was entered into a linear mixed effects model (“lmer” in R), with fully factorial fixed effects of mean FDC, Process, Material-Type, Paradigm, and Hemisphere. A single random intercept across participants was used. Results were reported in terms of Type III ANOVA using Satterthwaite’s method to adjust degrees of freedom. We then iterated this analysis, separately for Process (Recollection, Familiarity: p-corr(2)), and Process-and-Material-Type (Faces/Scenes/Words x Recollection/Familiarity: p-corr(6)).

Finally, comparisons with healthy controls on diencephalic volumes and mean FDC per tract were iterated for the subgroup of *n* = 14 LGI1-aLE patients (Argyropoulos et al. 2019) in our cohort. Comparisons with other autoantibody-related subgroups were not meaningful or feasible (S-Methods 1).

## Results

**Figure 1:**
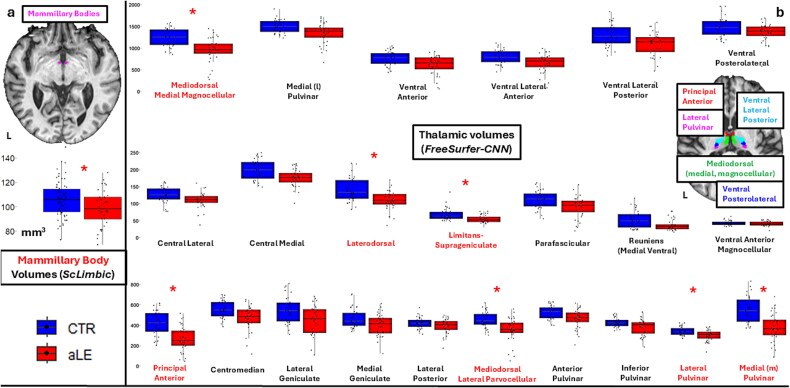
Volumes for a) mammillary bodies and b) thalamic nuclei; **key:** lines in boxplots = medians; bottom of box = 25th %ile; top of box = 75th %ile; upper, lower whiskers = scores outside the middle 50; whiskers = 1.5 × interquartile range; *: p-corr(Holm 1979) < 0.05; aLE: autoimmune limbic encephalitis patients; CTR: healthy controls; L/R: Left/Right (Hemisphere).

Our cohort showed volumetric reduction in the mammillary bodies (7%; *η^2^_p_* = 0.09), the principal anterior nuclei (36%; *η^2^_p_* = 0.33), the medial (m) (31%; *η^2^_p_* = 0.32) and lateral pulvinar (13%; *η^2^_p_* = 0.15), the medial mediodorsal (22%; *η^2^_p_* = 0.23) and lateral mediodorsal nuclei (22%; *η^2^_p_* = 0.19), but also the laterodorsal (21%; *η^2^_p_* = 0.17) and limitans-suprageniculate segmentation (20%; *η^2^_p_* = 0.14; all ANCOVAs: p-corr(24) < 0.05; Fig. 1; Table S2). These differences were conceptually replicated with HIPS-THOMAS and HypothalamicSubunits (Table S3).

**Figure 2:**
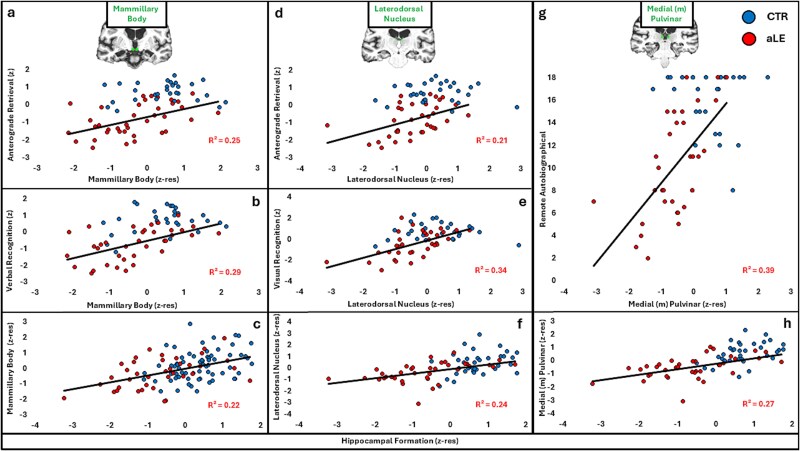
a–c: Relationships of mammillary body volumes with a) anterograde retrieval composite score, b) verbal recognition composite score, and c) hippocampal formation volume; d–f: relationships of laterodorsonal nucleus volumes with d) anterograde retrieval composite score, e) visual recognition, and f) hippocampal formation volume; g–h: relationships of medial (m) pulvinar volume with g) remote autobiographical memory and h) hippocampal formation volume. Data from healthy controls are not used in the calculation of the R^2^; **key:** aLE: autoimmune limbic encephalitis patients; CTR: healthy controls; z-res: standardized residuals.

Of the affected (*n* = 8) nuclei, the laterodorsal, medial (m) pulvinar, and mammillary bodies correlated with the volume of the manually delineated hippocampal formation (r = 0.47–0.52, p-corr(8) = 0.007–0.016; Fig. 2f–h). Of these 8 diencephalic nuclei, the mammillary bodies and the laterodorsal nuclei showed a volumetric relationship with anterograde retrieval (Fig. 2a and b), while remote autobiographical memory was associated with the volumes of the laterodorsal, limitans-suprageniculate, and medial (m) pulvinar segmentations (Fig. 2c; r = 0.46–0.62, p-corr(16) < 0.05; rest: r = 0.19–0.43, p-corr(16) ≥ 0.137). None of these volumes was associated with the semantic aspect of remote memories (r ≤ 0.18; *P* ≥ 0.339). We then iterated these analyses, after decomposing the anterograde composite score into verbal/visual recall/recognition scores, also examining hemisphere-specific volumes.

**Verbal recognition memory** correlated with mammillary body volumes (r = 0.54, p-corr(40) = 0.021; Fig. 2d; rest: r ≤ 0.39; p-corr(40) ≥ 0.485), driven by the left mammillary body (r = 0.61, p-corr(80) = 0.006; rest: r ≤ 0.43; p-corr(80) ≥ 0.497). While this volume correlated, at uncorrected levels, with verbal/visual recall and visual recognition scores (r = 0.41–0.53, *P* < 0.05, p-corr(80) ≥ 0.054), partial correlation analyses showed the relationship with verbal recognition held over and above visual recall/recognition (r = 0.41–0.45, *P* = 0.008–0.016), and, marginally, verbal recall (r = 0.30, *P* = 0.075). **Visual recognition memory** correlated with the volume of the laterodorsal nucleus (r = 0.58, p-corr(40) = 0.009; Fig. 2e), and marginally with medial (m) and lateral pulvinar volumes (r = 0.49–0.51, p-corr(40) = 0.064–0.086; rest: r ≤ 0.42, p-corr(40) ≥ 0.346). The relationship with the laterodorsal nucleus held over and above those with the medial (m) and lateral pulvinar (r = 0.35–0.42, *P* = 0.015–0.040). This was driven by the left laterodorsal nucleus (r = 0.60, p-corr(80) = 0.012; rest: r ≤ 0.52, p-corr(80) ≥ 0.107). While this volume also correlated with visual/verbal recall and verbal recognition (r = 0.43–0.51, *P* = 0.001–0.008), its links with visual recognition held over and above the rest (r = 0.40–0.46, *P* = 0.009–0.019). The relationship of the medial (m) pulvinar volume with **remote autobiographical memory** was driven by the left medial (m) pulvinar (r = 0.65, p-corr(80) = 0.004; left limitans-suprageniculate: r = 0.58, p-corr(80) = 0.031; right medial (m) pulvinar: r = 0.57, p-corr(80) = 0.046; left laterodorsal nucleus: r = 0.55, p-corr(80) = 0.075; rest: r ≤ 0.52, p-corr(80) ≥ 0.131). Its link with remote autobiographical memory held over and above these volumes (r ≥ 0.38; *P* ≤ 0.033). Importantly, these diencephalic relationships with memory held over and above (partial correlation) the total volume of the manually delineated hippocampal formation, as well as the strongest observable correlations of hippocampal/subicular subfield volumes with memory scores; in fact, diencephalic integrity fully mediated the effects of hippocampal volumes on memory scores (Table S4). No relationships between diencephalic volumes and **verbal/visual recall** survived correction(|r| ≤ 0.53; p-corr(80) ≥ 0.054).

**Figure 3:**
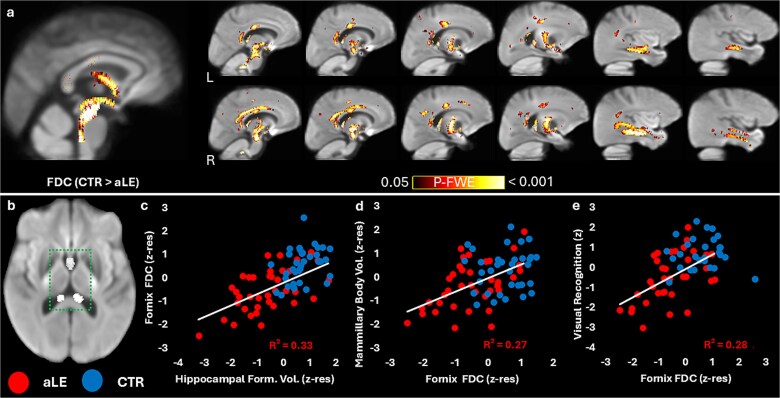
a) Fixel-based analysis comparisons between controls and patients on FDC. Clusters survive whole-brain threshold-free cluster-enhancement-correction (p-FWE < 0.05); b) binary fornix fixel mask; c–e: relationship of mean fornical FDC across patients with c) hippocampal formation volumes, d) mammillary body volumes, and e) visual recognition memory composite scores (Argyropoulos et al. 2019) (healthy control data are not used in the calculation of the R^2^); **key:** aLE: autoimmune limbic encephalitis; CTR: healthy controls; FDC: Fiber Density and Cross-section; L/R: eft/right hemisphere; z-res: standardized residuals.

A whole-brain fixel-based analysis showed reduced FDC in the fornix, cingulum, mammillothalamic tract, and internal capsule—all key components of the revised Papez circuit (Aggleton et al. 2022). However, abnormalities extended to the anterior commissure, the corpus callosum and its radiations, and the superior cerebellar peduncle (Fig. 3a). These were broadly replicated with voxel-based morphometry, tract-based spatial statistics, and tract reconstruction using deterministic tractography (Figs S2–S4).

Of the tracts compared between groups and found to show reduced FDC in patients (*n* = 25; Table S5), the fornix showed the largest effect size (η^2^_p_ = 0.32), followed by the superior cerebellar peduncle (η^2^_p_ = 0.32; rest: η^2^_p_ = 0.11–0.21). The fornix was also the only tract with reduced FDC to show a relationship, across patients, with hippocampal (r = 0.57, p-corr(50) = 0.011) and mammillary body volumes (r = 0.52, p-corr(50) = 0.048; rest: |r| < 0.48, p-corr(50) > 0.132; Fig. 3c-d). Corroborating the idea that these abnormalities occur as knock-on effects following hippocampal damage (Argyropoulos et al. 2019), the relationship between the hippocampal formation and the mammillary bodies was at least partly mediated by the integrity of the fornix (Fig. S5).

No relationship between mean tract FDC and the two composite scores (anterograde retrieval, remote autobiographical memory) survived correction for multiple tests (|r| < 0.53, p-corr(50) < 0.080), and the integrity of the fornix correlated with these scores only at uncorrected levels (remote autobiographical memory: r = 0.36, *P* = 0.039; anterograde retrieval: r = 0.35, p-unc = 0.034). The relationship with the latter was driven by visual recognition (r = 0.53, *P* = 0.001; Fig. 3e; verbal recognition: r = 0.30, *P* = 0.071; verbal recall: r = 0.34, *P* = 0.042), which held over and above verbal recall (r = 0.43, *P* = 0.012), verbal recognition (r = 0.47, *P* = 0.005), visual recall (r = 0.51, *P* = 0.002), and remote autobiographical memory (r = 0.40, *P* = 0.027). This pattern was corroborated by a series of exploratory analyses on the behavioral data from 8/38 aLE patients who had participated in custom-made recognition memory tasks dissociating recollection from familiarity (Argyropoulos et al. 2022). Fornical FDC was associated with familiarity for topographical scenes, not with face or word familiarity, or recollection (Table S6).

Consistent with the whole cohort, the subgroup (*n* = 14/38) of LGI1-aLE patients showed atrophy in the anterior, mediodorsal, and pulvinar nuclei, and compromised WM integrity in the fornix and beyond (Tables S7, S8, S9).

## Discussion

In a large patient cohort (*n* = 38) characterized by hippocampal atrophy and residual episodic memory impairment, we showed compromised integrity in specific diencephalic nuclei and WM tracts. These abnormalities explained aspects of amnesia over and above hippocampal volumes.

While whole-thalamic structural abnormalities have been demonstrated in aLE, and even specifically in LGI1-aLE (Argyropoulos et al. 2019; Qiao et al. 2025), there is hardly any evidence for nucleus-specific abnormalities in this condition. Here, the extent of the anterior thalamic atrophy (21%–36%) was comparable to, if not larger than that of the manually delineated hippocampal formation (22%–25%) (Argyropoulos et al. 2019), or the hippocampal-subicular subfields (15%–26%). Despite the evidence for atrophy in the anterior thalamus in medial temporal lobe epilepsy (Mueller et al. 2010; Tung et al. 2022) and its role in memory (de Bourbon-Teles et al. 2014; Sweeney-Reed et al. 2021; Aggleton et al. 2022), no relationships survived multiple-testing correction. This may reflect false negatives, lack of granularity in the segmentation of the anterior principal nuclei, or stronger involvement of the anteromedial and anterodorsal nuclei.

Mediodorsal atrophy (10%–22%) is also seen in temporal lobe epilepsy (Turski et al. 1986; Shimosaka et al. 1992). Evidence for projections from the perirhinal cortex and the adjacent amygdala (Graff-Radford et al. 1990; Parkin et al. 1994), task-based fMRI studies (Kafkas and Montaldi 2014), and neuropsychological research (Carlesimo et al. 2011) advocate for dissociations between mediodorsal and anterior nuclei in familiarity vs. recollection, respectively. However, we found no relationships with memory scores, and there is growing recognition that these nuclei primarily support executive aspects of memory (Mair et al. 2015; Perry et al. 2021; Wolff and Halassa 2024).

Atrophy in the laterodorsal nucleus (~21%) dovetails with its similarities in neocortical connectivity with the anterior thalamic nuclei (Robertson and Kaitz 1981; Jones 2012) and its inclusion in the anterior nuclear complex (Morel et al. 1997; Krauth et al. 2010). Consistent with its volumetric relationship with the hippocampal formation here, it has direct interconnections with the hippocampal formation (Aggleton et al. 1986; Van Groen and Wyss 1992; Vertes et al. 2007; Varela et al. 2014). Like the anterior thalamic nuclei, it is reciprocally connected with the posterior cingulate (Horikawa et al. 1988; Aggleton et al. 2014), wherein our patients showed reduced resting-state activity (Argyropoulos et al. 2019). Its volume predicted visual recognition memory, over and above any hippocampal-subicular subfield volumes. This aligns with its proposed involvement in visuospatial memory and the head direction signals it provides to the hippocampal formation (Warburton et al. 1997; van Groen et al. 2002; Perry and Mitchell 2019). Less is known about it in humans (Saunders et al. 2005; Cipolotti et al. 2008), or its role within the extended hippocampal system (Aggleton and Brown 1999; Aggleton et al. 2010; Aggleton et al. 2022). Its relationship with visual recognition memory over and above visual recall appears consistent with evidence that it contributes to familiarity (Cipolotti et al. 2008). However, our recognition tests involved photographs of doors and topographical scenes, whereas recall involved arbitrary shapes—the difference may thus pertain to material-type, rather than process—something which our data do not suffice to disambiguate.

We also saw medial (m) pulvinar atrophy (~31%). Whereas the rest of the pulvinar mainly connects with parietal and occipital cortices (Gutierrez et al. 2000), the medial pulvinar is reciprocally connected with the temporal lobe (Burton and Jones 1976; Mauguière and Baleydier 1978; Yeterian and Pandya 1985; Insausti et al. 1987; Baleydier and Morel 1992; Webster et al. 1993). Apart from perirhinal and entorhinal projections to the medial pulvinar via the temporopulvinar bundle (Saunders et al. 2005), the non-fornical projection from the subicular cortex to the laterodorsal nucleus passes through the medial pulvinar, with some additional termination there (Aggleton et al. 1986). In humans, the medial pulvinar is strongly connected (structurally, functionally) with the hippocampus (Guye et al. 2006; Rosenberg et al. 2006; Rosenberg et al. 2009; Catenoix et al. 2011; Morgan et al. 2015; Voets et al. 2015; Zheng et al. 2018; Segobin et al. 2019; Capecchi et al. 2020; Soulier et al. 2023) and the posterior cingulate (Baleydier and Mauguiere 1985; Shibata and Yukie 2003; Parvizi et al. 2006), wherein our patients showed reduced resting-state activity. It has also shown strong involvement in genetic frontotemporal dementia (Soskic et al. 2025), and, consistent with the strong link we observed with remote autobiographical memory, it is embedded within the default-mode network (Li et al. 2021), with its integrity being crucial for autobiographical retrieval (Philippi et al. 2015).

Despite our study’s cross-sectional nature, the evidence above and our mediation analyses support the possibility that these abnormalities reflect knock-on effects, akin to those in animal models (Machado et al. 2008; Meng et al. 2014; Meng et al. 2016). Of the few longitudinal MRI studies on aLE, Wagner et al. (2015) reported volume reduction from the acute to the chronic stage in the medial temporal lobe, the basal ganglia, but also the thalamus, albeit with no further details.

Given its connectivity with the hippocampal formation and the prefrontal cortex (Yanagihara et al. 1985; Vertes 2006; Vertes et al. 2007; Varela et al. 2014), the absence of atrophy in the medioventral–reuniens segmentation is hard to interpret, as is the reduction in the limitans-suprageniculate. Despite the evidence for the connectivity of the latter with the prefrontal (Preuss and Goldman-Rakic 1987; Burman et al. 2011) and perirhinal cortices (Kimura et al. 2003; Tomás Pereira et al. 2016), there is little information on abnormalities in hippocampal damage (Lee et al. 2020) or on its involvement in memory (Kim et al. 2020). Given the very small volumes of these two segmentations, and that they have only been generated with FreeSurfer-CNN, replication is required in other cohorts.

All WM analyses disclosed abnormalities in the fornix, complementing the atrophy in the mammillary bodies and the principal anterior nuclei—projections to the latter almost exclusively involve the fornix (Saunders et al. 2005). Of the affected tracts, mammillary body volumes exclusively correlated with the fornix, consistent with recent work in unilateral mesial temporal sclerosis (Mojica et al. 2026). A mediation analysis supported the plausibility of a causal link, whereby hippocampal damage compromises the fornix, which in turn compromises the mammillary bodies. There is evidence consistent with Wallerian degeneration of the fornix following hippocampal damage (Baldwin et al. 1994; Fernandez et al. 2022) and of the mammillary bodies following fornical transections (Zola-Morgan et al. 1989; Loftus et al. 2000), while fornix damage does not produce retrograde degeneration in its fibers from the temporal lobe (Daitz and Powell 1954; Saunders and Aggleton 2007). Mammillary body volumes correlated strongly with verbal recognition, over and above hippocampal volumes and, marginally, verbal recall. Correlations of fornical integrity (FDC) with memory scores were only observed at uncorrected levels, and only with visual recognition memory (Argyropoulos et al. 2019) and familiarity estimates for topographical scenes (Argyropoulos et al. 2022). This is not consistent with some studies in healthy participants (Nestor et al. 2007; Rudebeck et al. 2009; Hartopp et al. 2019; Metzler-Baddeley et al. 2019; Coad et al. 2020) or patients (Carlesimo et al. 2007; Tsivilis et al. 2008; Vann et al. 2009) showing selective involvement of the fornix/mammillary bodies in recall/recollection (Aggleton and Brown 1999; Montaldi and Mayes 2010; Aggleton et al. 2023—however, see: Calabrese et al. 1995; D’Esposito et al. 1995; Park et al. 2000; Gold and Squire 2006; Poreh et al. 2006; Cipolotti et al. 2008). Regardless, our findings do not necessarily reflect a replication failure, given substantial differences in etiological and neuropathological profiles.

Abnormalities in the cingulum (captured with all methods) are consistent with the atrophy in the anterior thalamic nuclei, since the cingulum involves projections of these nuclei to the hippocampal formation (Aggleton et al. 2022). They also dovetail with the reduced resting-state activity and hippocampal connectivity seen in the cingulate and retrosplenial cortices (Argyropoulos et al. 2019), since the cingulum comprises reciprocal connections between the anterior thalamic nuclei and the cingulate and retrosplenial cortices (Domesick 1970; Shibata 1993a; Shibata 1993b; Heilbronner and Haber 2014; Bubb et al. 2018; Bubb et al. 2020).

Abnormalities were also seen in the mammillothalamic tract and the internal capsule. The latter carries projections from the hippocampal formation and entorhinal cortex to the principal anterior and laterodorsal nuclei (Aggleton et al. 1986; Saunders et al. 2005; Dillingham et al. 2015), both of which were atrophied in our patients.

In agreement with recent evidence for whole-brain structural connectivity and aberrant resting-state functional connectivity across several large-scale networks in LGI1-aLE (Heine et al. 2018; Qiao et al. 2020; Krohn et al. 2025), we saw abnormalities beyond the hippocampal-diencephalic-cingulate network, especially in the corpus callosum and its radiations, the superior cerebellar peduncle, and the external capsule. The last two dovetail with the atrophic mediodorsal thalamus: the lateral cerebellar nucleus reaches the lateral mediodorsal nucleus via the superior cerebellar peduncle (Çavdar et al. 2014), and the external capsule carries entorhinal-perirhinal projections to the mediodorsal nuclei (Saunders et al. 2005). Callosal abnormalities have been reported in non-human primates following hippocampal damage (Meng et al. 2014; Payne et al. 2017), and in patients with hippocampal sclerosis (Raffelt et al. 2017). Although the hippocampus does not send inter-hemispheric projections via the corpus callosum, the cortical regions that are interconnected with the hippocampus do (Meng et al. 2014; Meng et al. 2018).

Even so, we acknowledge that our study cannot discard the possibility that changes in thalamic integrity are not due to Wallerian degeneration, but a direct effect of antibodies within the brain. LGI1 is known to be expressed strongly in the hippocampus (Herranz-Pérez et al. 2010; Ohkawa et al. 2013). However, partly due to methodological differences, other studies have disclosed more widespread distribution (Ramirez-Franco et al. 2022), wherein the thalamus has been noted—albeit not the nuclei discussed here (Schulte et al. 2006; Silva et al. 2011; Zhou et al. 2012).

Overall, our work urges caution in assuming “focal hippocampal damage”, even in patients with conditions studied as “human models” of such damage. It also underscores the possibility that network-wide spread of pathology may occur as a knock-on effect of hippocampal damage, such as that in specific diencephalic nuclei (even beyond the principal anterior nuclei or the mammillary bodies), and may strongly predict amnesia, over and above hippocampal integrity.

## Supplementary Material

CerCor_supplementary_06_26_AmendedUpload(13)

## Data Availability

Demographic, behavioral, and volumetric data are publicly available at https://osf.io/5ybnk/

## References

[ref1] Aggleton JP, Brown MW. 1999. Episodic memory, amnesia, and the hippocampal–anterior thalamic axis. Behav Brain Sci. 22:425–444. 10.1017/S0140525X99002034. 11301518

[ref2] Aggleton JP, Desimone R, Mishkin M. 1986. The origin, course, and termination of the hippocampothalamic projections in the macaque. J Comp Neurol. 243:409–421. 10.1002/cne.902430310. 3512627

[ref3] Aggleton JP, Saunders RC, Vann SD. 2008. Chapter 5.2 using hippocampal amnesia to understand the neural basis of diencephalic amnesia. In: Handbook of Behavioral neuroscience Dere E, Easton A, Nadel L, Huston JP (eds), Vol. 18. Elsevier, pp 503–632 (Handbook of Episodic Memory). [accessed 2024 July 22]. https://www.sciencedirect.com/science/article/pii/S1569733908002270.

[ref4] Aggleton JP et al. 2010. Hippocampal-anterior thalamic pathways for memory: uncovering a network of direct and indirect actions. Eur J Neurosci. 31:2292–2307. 10.1111/j.1460-9568.2010.07251.x. 20550571 PMC2936113

[ref5] Aggleton JP, Saunders RC, Wright NF, Vann SD. 2014. The origin of projections from the posterior cingulate and retrosplenial cortices to the anterior, medial dorsal and laterodorsal thalamic nuclei of macaque monkeys. Eur J Neurosci. 39:107–123. 10.1111/ejn.12389. 24134130 PMC4112842

[ref6] Aggleton JP, Nelson AJD, O’Mara SM. 2022. Time to retire the serial Papez circuit: implications for space, memory, and attention. Neurosci Biobehav Rev. 140:104813. 10.1016/j.neubiorev.2022.104813. 35940310 PMC10804970

[ref7] Aggleton JP, Vann SD, O’Mara SM. 2023. Converging diencephalic and hippocampal supports for episodic memory. Neuropsychologia. 191:108728. 10.1016/j.neuropsychologia.2023.108728. 37939875

[ref8] Andica C et al. 2021. Fiber-specific white matter alterations in early-stage tremor-dominant Parkinson’s disease. NPJ Parkinsons Dis. 7:51. 10.1038/s41531-021-00197-4. 34172728 PMC8233424

[ref9] Argyropoulos GPD, Butler CR. 2020. Does hippocampal atrophy explain anterograde and retrograde amnesia following autoimmune limbic encephalitis? Hippocampus. 30:1013–1017. 10.1002/hipo.23208. 32320116

[ref10] Argyropoulos GPD et al. 2019. Network-wide abnormalities explain memory variability in hippocampal amnesia. elife. 8:e46156. 10.7554/eLife.46156. 31282861 PMC6639076

[ref11] Argyropoulos GPD et al. 2020. Pathologic tearfulness after limbic encephalitis: a novel disorder and its neural basis. Neurology. 94:e1320–e1335. 10.1212/WNL.0000000000008934. 31980582 PMC7274928

[ref12] Argyropoulos GPD et al. 2022. Functional specialization of the medial temporal lobes in human recognition memory: dissociating effects of hippocampal versus Parahippocampal damage. Cereb Cortex. 32:1637–1652. 10.1093/cercor/bhab290. 34535797 PMC9016283

[ref13] Argyropoulos GPD, Butler CR, Saranathan M . 2025. Toward Reliable Thalamic Segmentation: a rigorous evaluation of automated methods for structural MRI. medRxiv, 2025.09.09: 25335439. 10.1101/2025.09.09.25335439. PMC13380577 42470458

[ref14] Baddeley A, Emslie H, Nimmo-Smith I. 1994. Doors and people: a test of visual and verbal recall and recognition. Thames Valley Test Co.

[ref15] Baldwin GN, Tsuruda JS, Maravilla KR, Hamill GS, Hayes CE. 1994. The fornix in patients with seizures caused by unilateral hippocampal sclerosis: detection of unilateral volume loss on MR images. Am J Roentgenol. 162:1185–1189. 10.2214/ajr.162.5.8166008. 8166008

[ref16] Baleydier C, Mauguiere F. 1985. Anatomical evidence for medial pulvinar connections with the posterior cingulate cortex, the retrosplenial area, and the posterior parahippocampal gyrus in monkeys. J Comp Neurol. 232:219–228. 10.1002/cne.902320207. 3973091

[ref17] Baleydier C, Morel A. 1992. Segregated thalamocortical pathways to inferior parietal and inferotemporal cortex in macaque monkey. Vis Neurosci. 8:391–405. 10.1017/S0952523800004922. 1375095

[ref18] Baron RM, Kenny DA. 1986. The moderator–mediator variable distinction in social psychological research: conceptual, strategic, and statistical considerations. J Pers Soc Psychol. 51:1173–1182. 10.1037/0022-3514.51.6.1173. 3806354

[ref19] Basile BM, Templer VL, Gazes RP, Hampton RR. 2020. Preserved visual memory and relational cognition performance in monkeys with selective hippocampal lesions. Sci Adv. 6:eaaz0484. 10.1126/sciadv.aaz0484. 32832615 PMC7439495

[ref20] Bastiaansen AEM, van Sonderen A, Titulaer MJ. 2017. Autoimmune encephalitis with anti-leucine-rich glioma-inactivated 1 or anti-contactin-associated protein-like 2 antibodies (formerly called voltage-gated potassium channel-complex antibodies). Curr Opin Neurol. 30:302–309. 10.1097/WCO.0000000000000444. 28248701

[ref21] Benear SL, Ngo CT, Olson IR. 2020. Dissecting the fornix in basic memory processes and neuropsychiatric disease: a review. Brain Connect. 10:331–354. 10.1089/brain.2020.0749. 32567331 PMC7495920

[ref22] Billot B et al. 2020. Automated segmentation of the hypothalamus and associated subunits in brain MRI. NeuroImage. 223:117287. 10.1016/j.neuroimage.2020.117287. 32853816 PMC8417769

[ref23] Bird CM, Burgess N. 2008. The hippocampus supports recognition memory for familiar words but not unfamiliar faces. Curr Biol. 18:1932–1936. 10.1016/j.cub.2008.10.046. 19084409

[ref24] de Bourbon-Teles J et al. 2014. Thalamic control of human attention driven by memory and learning. Curr Biol. 24:993–999. 10.1016/j.cub.2014.03.024. 24746799 PMC4012133

[ref25] Bubb EJ, Kinnavane L, Aggleton JP. 2017. Hippocampal - diencephalic - cingulate networks for memory and emotion: an anatomical guide. Brain Neurosci Adv. 1:2398212817723443. 10.1177/2398212817723443. PMC5608081 28944298

[ref26] Bubb EJ, Metzler-Baddeley C, Aggleton JP. 2018. The cingulum bundle: anatomy, function, and dysfunction. Neurosci Biobehav Rev. 92:104–127. 10.1016/j.neubiorev.2018.05.008. 29753752 PMC6090091

[ref27] Bubb EJ, Nelson AJD, Cozens TC, Aggleton JP. 2020. Organisation of cingulum bundle fibres connecting the anterior thalamic nuclei with the rodent anterior cingulate and retrosplenial cortices. Brain Neurosci Adv. 4:239821282095716. 10.1177/2398212820957160. PMC7488606 32964131

[ref28] Burman KJ et al. 2011. Subcortical projections to the frontal pole in the marmoset monkey. Eur J Neurosci. 34:303–319. 10.1111/j.1460-9568.2011.07744.x. 21714814

[ref29] Burton H, Jones EG. 1976. The posterior thalamic region and its cortical projection in new world and old world monkeys. J Comp Neurol. 168:249–301. 10.1002/cne.901680204. 821975

[ref30] Calabrese P, Markowitsch HJ, Harders AG, Scholz M, Gehlen W. 1995. Fornix damage and memory: a case report. Cortex. 31:555–564. 10.1016/S0010-9452(13)80066-4. 8536482

[ref31] Capecchi F, Mothersill I, Imbach LL. 2020. The medial pulvinar as a subcortical relay in temporal lobe status epilepticus. Seizure - European Journal of Epilepsy. 81:276–279. 10.1016/j.seizure.2020.08.016. 32919252

[ref32] Carlesimo GA et al. 2007. Bilateral damage to the mammillo-thalamic tract impairs recollection but not familiarity in the recognition process: a single case investigation. Neuropsychologia. 45:2467–2479. 10.1016/j.neuropsychologia.2007.03.025. 17512561

[ref33] Carlesimo GA, Lombardi MG, Caltagirone C. 2011. Vascular thalamic amnesia: a reappraisal. Neuropsychologia. 49:777–789. 10.1016/J.NEUROPSYCHOLOGIA.2011.01.026. 21255590

[ref34] Catenoix H, Magnin M, Mauguière F, Ryvlin P. 2011. Evoked potential study of hippocampal efferent projections in the human brain. Clin Neurophysiol. 122:2488–2497. 10.1016/j.clinph.2011.05.007. 21669549

[ref35] Çavdar S, Özgür M, Uysal SP, Amuk ÖC. 2014. Motor afferents from the cerebellum, zona incerta and substantia nigra to the mediodorsal thalamic nucleus in the rat. J Integr Neurosci. 13:565–578. 10.1142/S0219635214500198. 25164360

[ref36] Cipolotti L et al. 2008. The role of the thalamus in amnesia: a tractography, high-resolution MRI and neuropsychological study. Neuropsychologia. 46:2745–2758. 10.1016/j.neuropsychologia.2008.05.009. 18597798

[ref37] Clarke S et al. 1994. Pure amnesia after unilateral left polar thalamic infarct: topographic and sequential neuropsychological and metabolic (PET) correlations. J Neurol Neurosurg Psychiatry. 57:27–34. 10.1136/jnnp.57.1.27. 8301301 PMC485036

[ref38] Coad BM et al. 2020. Precommissural and postcommissural fornix microstructure in healthy aging and cognition. Brain Neurosci Adv. 4:239821281989931. 10.1177/2398212819899316. PMC7085915 32219177

[ref39] Cormack F et al. 2005. Extra-hippocampal grey matter density abnormalities in paediatric mesial temporal sclerosis. NeuroImage. 27:635–643. 10.1016/j.neuroimage.2005.05.023. 16006149

[ref40] D’Esposito M, Verfaellie M, Alexander MP, Katz DI. 1995. Amnesia following traumatic bilateral fornix transection. Neurology. 45:1546–1550. 10.1212/wnl.45.8.1546. 7644056

[ref41] Daitz HM, Powell TP. 1954. Studies of the connexions of the fornix system. J Neurol Neurosurg Psychiatry. 17:75–82. 10.1136/jnnp.17.1.75. 13131081 PMC503161

[ref42] Damasio AR, Van Hoesen GW. 1985. The limbic system and the localisation of herpes simplex encephalitis. J Neurol Neurosurg Psychiatry. 48:297–301. 10.1136/jnnp.48.4.297. 3998736 PMC1028292

[ref43] Dede AJO, Wixted JT, Hopkins RO, Squire LR. 2016a. Autobiographical memory, future imagining, and the medial temporal lobe. Proc Natl Acad Sci. 113:13474–13479. 10.1073/pnas.1615864113. 27821735 PMC5127323

[ref44] Dede AJO, Frascino JC, Wixted JT, Squire LR. 2016b. Learning and remembering real-world events after medial temporal lobe damage. Proc Natl Acad Sci. 113:13480–13485. 10.1073/pnas.1617025113. 27821761 PMC5127365

[ref45] Dillingham CM, Erichsen JT, O'Mara SM, Aggleton JP, Vann SD. 2015. Fornical and nonfornical projections from the rat hippocampal formation to the anterior thalamic nuclei. Hippocampus. 25:977–992. 10.1002/hipo.22421. 25616174 PMC4737193

[ref46] Domesick VB . 1970. The fasciculus cinguli in the rat. Brain Res. 20:19–32. 10.1016/0006-8993(70)90150-2. 5444766

[ref47] Dunstan EJ, Winer JB. 2006. Autoimmune limbic encephalitis causing fits, rapidly progressive confusion and hyponatraemia. Age Ageing. 35:536–537. 10.1093/ageing/afl045. 16763057

[ref48] Dzieciol AM et al. 2017. Hippocampal and diencephalic pathology in developmental amnesia. Cortex. 86:33–44 (Is a “single” brain model sufficient?). 10.1016/j.cortex.2016.09.016. 27880886 PMC5264402

[ref49] Elliott CA, Gross DW, Wheatley BM, Beaulieu C, Sankar T. 2018. Longitudinal hippocampal and extra-hippocampal microstructural and macrostructural changes following temporal lobe epilepsy surgery. Epilepsy Res. 140:128–137. 10.1016/j.eplepsyres.2018.01.008. 29331847

[ref50] Fernandez AM, Gutekunst CA, Grogan DP, Pedersen NP, Gross RE. 2022. Loss of efferent projections of the hippocampal formation in the mouse intrahippocampal kainic acid model. Epilepsy Res. 180:106863. 10.1016/j.eplepsyres.2022.106863. 35114430

[ref51] Finke C et al. 2017. Evaluation of cognitive deficits and structural hippocampal damage in encephalitis with leucine-rich, glioma-inactivated 1 antibodies. JAMA Neurol. 74:50–59. 10.1001/jamaneurol.2016.4226. 27893017

[ref52] Gitelman DR, Ashburner J, Friston KJ, Tyler LK, Price CJ. 2001. Voxel-based morphometry of herpes simplex encephalitis. NeuroImage. 13:623–631. 10.1006/nimg.2000.0734. 11305891

[ref53] Gold JJ, Squire LR. 2006. The anatomy of amnesia: Neurohistological analysis of three new cases. Learn Mem. 13:699–710. 10.1101/lm.357406. 17101872 PMC1783623

[ref54] Gold JJ et al. 2006. Item memory, source memory, and the medial temporal lobe: concordant findings from fMRI and memory-impaired patients. Proc Natl Acad Sci USA. 103:9351–9356. 10.1073/pnas.0602716103. 16751272 PMC1482613

[ref55] Graff-Radford NR, Tranel D, Van Hoesen GW, Brandt JP. 1990. Diencephalic amnesia. Brain. 113:1–25. 10.1093/brain/113.1.1. 2302527

[ref56] Graus F et al. 2016. A clinical approach to diagnosis of autoimmune encephalitis. Lancet Neurol. 15:391–404. 10.1016/S1474-4422(15)00401-9. 26906964 PMC5066574

[ref57] Greve DN et al. 2021. A deep learning toolbox for automatic segmentation of subcortical limbic structures from MRI images. NeuroImage. 244:118610. 10.1016/j.neuroimage.2021.118610. 34571161 PMC8643077

[ref59] Gross DW . 2011. Diffusion tensor imaging in temporal lobe epilepsy. Epilepsia. 52:32–34. 10.1111/j.1528-1167.2011.03149.x. 21732939

[ref60] Gutierrez C, Cola MG, Seltzer B, Cusick C. 2000. Neurochemical and connectional organization of the dorsal pulvinar complex in monkeys. J Comp Neurol. 419:61–86. 10.1002/(SICI)1096-9861(20000327)419:1<61::AID-CNE4>3.0.CO;2-I. 10717640

[ref61] Guye M et al. 2006. The role of corticothalamic coupling in human temporal lobe epilepsy. Brain. 129:1917–1928. 10.1093/brain/awl151. 16760199

[ref62] Harms A et al. 2023. Mesiotemporal Volumetry, cortical thickness, and neuropsychological deficits in the long-term course of limbic encephalitis. Neurol Neuroimmunol Neuroinflamm. 10:e200125. 10.1212/NXI.0000000000200125. 37230543 PMC10211327

[ref63] Hartopp N et al. 2019. A key role for subiculum-fornix connectivity in recollection in older age. Front Syst Neurosci. 12:70. 10.3389/fnsys.2018.00070. PMC6335321 30687030

[ref64] Hassabis D, Kumaran D, Vann SD, Maguire EA. 2007. Patients with hippocampal amnesia cannot imagine new experiences. Proc Natl Acad Sci USA. 104:1726–1731. 10.1073/pnas.0610561104. 17229836 PMC1773058

[ref65] Hayes SM, Salat DH, Verfaellie M. 2012. Default network connectivity in medial temporal lobe amnesia. J Neurosci. 32:14622–14629a. 10.1523/JNEUROSCI.0700-12.2012. 23077048 PMC3573849

[ref66] Heilbronner SR, Haber SN. 2014. Frontal cortical and subcortical projections provide a basis for segmenting the cingulum bundle: implications for neuroimaging and psychiatric disorders. J Neurosci. 34:10041–10054. 10.1523/JNEUROSCI.5459-13.2014. 25057206 PMC4107396

[ref67] Heilman KM et al. 1990. Frontal hypermetabolism and thalamic hypometabolism in a patient with abnormal orienting and retrosplenial amnesia. Neuropsychologia. 28:161–169. 10.1016/0028-3932(90)90098-9. 2314571

[ref68] Heine J et al. 2018. Beyond the limbic system: disruption and functional compensation of large-scale brain networks in patients with anti-LGI1 encephalitis. J Neurol Neurosurg Psychiatry. 89:1191–1199. 10.1136/jnnp-2017-317780. 29886429

[ref69] Heinz UE, Rollnik JD. 2015. Outcome and prognosis of hypoxic brain damage patients undergoing neurological early rehabilitation. BMC Res Notes. 8:243. 10.1186/s13104-015-1175-z. 26081628 PMC4469251

[ref70] Henson RN et al. 2016. The effects of hippocampal lesions on MRI measures of structural and functional connectivity. Hippocampus. 26:1447–1463. 10.1002/hipo.22621. 27479794 PMC5082505

[ref71] Henson RN et al. 2017. No effect of hippocampal lesions on stimulus-response bindings. Neuropsychologia. 103:106–114. 10.1016/j.neuropsychologia.2017.07.024. 28739442 PMC5726084

[ref72] Herranz-Pérez V, Olucha-Bordonau FE, Morante-Redolat JM, Pérez-Tur J. 2010. Regional distribution of the leucine-rich glioma inactivated (*LGI*) gene family transcripts in the adult mouse brain. Brain Res. 1307:177–194. 10.1016/j.brainres.2009.10.013. 19833108

[ref73] Holm S . 1979. A simple sequentially rejective multiple test procedure. Scand J Stat. 6:65–70. 10.2307/4615733.

[ref74] Horikawa K, Kinjo N, Stanley LC, Powell EW. 1988. Topographic organization and collateralization of the projections of the anterior and laterodorsal thalamic nuclei to cingulate areas 24 and 29 in the rat. Neurosci Res. 6:31–44. 10.1016/0168-0102(88)90004-1. 3200518

[ref75] Huang BY, Castillo M. 2008. Hypoxic-ischemic brain injury: imaging findings from birth to adulthood. Radiographics. 28:417–439. 10.1148/rg.282075066. 18349449

[ref76] Iglesias JE et al. 2015. A computational atlas of the hippocampal formation using *ex vivo*, ultra-high resolution MRI: application to adaptive segmentation of *in vivo* MRI. NeuroImage. 115:117–137. 10.1016/j.neuroimage.2015.04.042. 25936807 PMC4461537

[ref77] Iglesias JE et al. 2018. A probabilistic atlas of the human thalamic nuclei combining ex vivo MRI and histology. NeuroImage. 183:314–326. 10.1016/j.neuroimage.2018.08.012. 30121337 PMC6215335

[ref78] Insausti R, Amaral DG, Cowan WM. 1987. The entorhinal cortex of the monkey: III. Subcortical afferents. J Comp Neurol. 264:396–408. 10.1002/cne.902640307. 3680636

[ref79] Jones EG . 2012. The thalamus. Springer Science & Business Media.

[ref80] Jonin P-Y et al. 2018. Superior explicit memory despite severe developmental amnesia: In-depth case study and neural correlates. Hippocampus. 28:867–885. 10.1002/hipo.23010. 29995351

[ref81] Judd CM, Kenny DA. 1981. Process analysis: estimating mediation in treatment evaluations. Eval Rev. 5:602–619. 10.1177/0193841X8100500502.

[ref82] Kafkas A, Montaldi D. 2014. Two separate, but interacting, neural systems for familiarity and novelty detection: a dual-route mechanism. Hippocampus. 24:516–527. 10.1002/hipo.22241. 24436072

[ref83] Kafkas A et al. 2017. Material specificity drives medial temporal lobe familiarity but not hippocampal recollection. Hippocampus. 27:194–209. 10.1002/hipo.22683. 27859925 PMC5299537

[ref84] Kafkas A, Mayes AR, Montaldi D. 2020. Thalamic-medial temporal lobe connectivity underpins familiarity memory. Cereb Cortex. 30:3827–3837. 10.1093/cercor/bhz345. 31989161 PMC7232995

[ref85] Kahhale I, Buser NJ, Madan CR, Hanson JL. 2023. Quantifying numerical and spatial reliability of hippocampal and amygdala subdivisions in FreeSurfer. Brain Inform. 10:9. 10.1186/s40708-023-00189-5. 37029203 PMC10082143

[ref86] Kelly MJ et al. 2024. Magnetic resonance imaging characteristics of LGI1-antibody and CASPR2-antibody encephalitis. JAMA Neurol. 81:525–533. 10.1001/jamaneurol.2024.0126. 38497971 PMC10949153

[ref87] Khan NL, Jeffree MA, Good C, Macleod W, al-Sarraj S. 2009. Histopathology of VGKC antibody-associated limbic encephalitis. Neurology. 72:1703–1705. 10.1212/WNL.0b013e3181a55eb3. 19433746

[ref88] Kim S, Dede AJO, Hopkins RO, Squire LR. 2015. Memory, scene construction, and the human hippocampus. Proc Natl Acad Sci USA. 112:4767–4772. 10.1073/pnas.1503863112. 25825712 PMC4403152

[ref89] Kim HF, Griggs WS, Hikosaka O. 2020. Long-term value memory in the primate posterior thalamus for fast automatic action. Curr Biol. 30:2901–2911.e3. 10.1016/j.cub.2020.05.047. 32531286 PMC9422328

[ref90] Kimura A, Donishi T, Sakoda T, Hazama M, Tamai Y. 2003. Auditory thalamic nuclei projections to the temporal cortex in the rat. Neuroscience. 117:1003–1016. 10.1016/S0306-4522(02)00949-1. 12654352

[ref91] Kopelman MD, Wilson BA, Baddeley AD. 1989. The autobiographical memory interview: a new assessment of autobiographical and personal semantic memory in amnesic patients. J Clin Exp Neuropsychol. 11:724–744. 10.1080/01688638908400928. 2808661

[ref92] Kopelman MD et al. 2007. Recall and recognition memory in amnesia: patients with hippocampal, medial temporal, temporal lobe or frontal pathology. Neuropsychologia. 45:1232–1246. 10.1016/j.neuropsychologia.2006.10.005. 17140609

[ref93] Krauth A et al. 2010. A mean three-dimensional atlas of the human thalamus: generation from multiple histological data. NeuroImage. 49:2053–2062. 10.1016/j.neuroimage.2009.10.042. 19853042

[ref94] Krohn S et al. 2025. Cognitive deficits in anti-LGI1 encephalitis are linked to immunotherapy-resistant white matter network changes. Neurol Neuroimmunol Neuroinflamm. 12:e200360. 10.1212/NXI.0000000000200360. 39879565 PMC11789668

[ref95] Lad M, Mullally SL, Houston AL, Kelly T, Griffiths TD. 2019. Characterizing memory loss in patients with autoimmune limbic encephalitis hippocampal lesions. Hippocampus. 29:1114–1120. 10.1002/hipo.23150. 31472008 PMC6852518

[ref96] Lee ACH et al. 2006. Differentiating the roles of the hippocampus and perirhinal cortex in processes beyond long-term declarative memory: a double dissociation in dementia. J Neurosci. 26:5198–5203. 10.1523/JNEUROSCI.3157-05.2006. 16687511 PMC6674247

[ref97] Lee ACH, Yeung L-K, Barense MD. 2012. The hippocampus and visual perception. Front Hum Neurosci. 6:91. 10.3389/fnhum.2012.00091. 22529794 PMC3328126

[ref98] Lee H-J, Seo SA, Park KM. 2020. Quantification of thalamic nuclei in patients diagnosed with temporal lobe epilepsy and hippocampal sclerosis. Neuroradiology. 62:185–195. 10.1007/s00234-019-02299-6. 31673749

[ref99] Li J et al. 2021. Mapping the subcortical connectivity of the human default mode network. NeuroImage. 245:118758. 10.1016/j.neuroimage.2021.118758. 34838949 PMC8945548

[ref100] Loftus M, Knight RT, Amaral DG. 2000. An analysis of atrophy in the medial mammillary nucleus following hippocampal and fornix lesions in humans and nonhuman primates. Exp Neurol. 163:180–190. 10.1006/exnr.2000.7361. 10785457

[ref101] Machado CJ, Snyder AZ, Cherry SR, Lavenex P, Amaral DG. 2008. Effects of neonatal amygdala or hippocampus lesions on resting brain metabolism in the macaque monkey: a microPET imaging study. NeuroImage. 39:832–846. 10.1016/j.neuroimage.2007.09.029. 17964814 PMC2527971

[ref102] MacKinnon DP, Fairchild AJ, Fritz MS. 2007. Mediation analysis. Annu Rev Psychol. 58:593–614. 10.1146/annurev.psych.58.110405.085542. 16968208 PMC2819368

[ref103] Maffei C et al. 2021. Using diffusion MRI data acquired with ultra-high gradient strength to improve tractography in routine-quality data. NeuroImage. 245:118706. 10.1016/j.neuroimage.2021.118706. 34780916 PMC8835483

[ref104] Maguire EA, Mullally SL. 2013. The hippocampus: a manifesto for change. J Exp Psychol Gen. 142:1180–1189. 10.1037/a0033650. 23855494 PMC3906798

[ref105] Maguire EA, Nannery R, Spiers HJ. 2006. Navigation around London by a taxi driver with bilateral hippocampal lesions. Brain. 129:2894–2907. 10.1093/brain/awl286. 17071921

[ref106] Maguire EA, Intraub H, Mullally SL. 2016. Scenes, spaces, and memory traces: what does the hippocampus do? Neuroscientist. 22:432–439. 10.1177/1073858415600389. 26276163 PMC5021215

[ref107] Mair RG et al. 2015. The neurobiology of thalamic amnesia: contributions of medial thalamus and prefrontal cortex to delayed conditional discrimination. Neurosci Biobehav Rev. 54:161–174 (The Cognitive Thalamus). 10.1016/j.neubiorev.2015.01.011. 25616180

[ref108] Mauguière F, Baleydier C. 1978. Topographical organization of medial pulvinar neurons sending fibres to Brodman’s areas 7, 21 and 22 in the monkey. Exp Brain Res. 31:605–607. 10.1007/BF00239815. 95961

[ref109] McCormick C, Rosenthal CR, Miller TD, Maguire EA. 2016. Hippocampal damage increases deontological responses during moral decision making. J Neurosci. 36:12157–12167. 10.1523/JNEUROSCI.0707-16.2016. 27903725 PMC5148217

[ref110] McCormick C, Rosenthal CR, Miller TD, Maguire EA. 2018. Mind-wandering in people with hippocampal damage. J Neurosci. 38:2745–2754. 10.1523/JNEUROSCI.1812-17.2018. 29440532 PMC5851780

[ref111] Meng Y et al. 2014. Alterations of hippocampal projections in adult macaques with neonatal hippocampal lesions: a diffusion tensor imaging study. NeuroImage. 102:828–837. 10.1016/j.neuroimage.2014.08.059. 25204865 PMC4252575

[ref112] Meng Y, Hu X, Bachevalier J, Zhang X. 2016. Decreased functional connectivity in dorsolateral prefrontal cortical networks in adult macaques with neonatal hippocampal lesions: relations to visual working memory deficits. Neurobiol Learn Mem. 134:31–37. 10.1016/j.nlm.2016.04.003. 27063864 PMC6329462

[ref113] Meng Y, Hu X, Zhang X, Bachevalier J. 2018. Diffusion tensor imaging reveals microstructural alterations in corpus callosum and associated transcallosal fiber tracts in adult macaques with neonatal hippocampal lesions. Hippocampus. 28:838–845. 10.1002/hipo.23006. 29978933 PMC6282702

[ref114] Metzler-Baddeley C et al. 2019. Fornix white matter glia damage causes hippocampal gray matter damage during age-dependent limbic decline. Sci Rep. 9:1060. 10.1038/s41598-018-37658-5. 30705365 PMC6355929

[ref115] Meys KME, de Vries LS, Groenendaal F, Vann SD, Lequin MH. 2022. The mammillary bodies: a review of causes of injury in infants and children. Am J Neuroradiol. 43:802–812. 10.3174/ajnr.A7463. 35487586 PMC9172959

[ref116] Miller TD et al. 2017. Focal CA3 hippocampal subfield atrophy following LGI1 VGKC-complex antibody limbic encephalitis. Brain. 140:1212–1219. 10.1093/brain/awx070. 28369215 PMC5405234

[ref117] Miller TD et al. 2020. Human hippocampal CA3 damage disrupts both recent and remote episodic memories Barense M, Colgin LL, Barense M, editors. elife. 9:e41836. 10.7554/eLife.41836. 31976861 PMC6980860

[ref118] Mojica M, Fleming H, Krings T, McAndrews MP. 2026. Hippocampal, fornix, and mammillary body atrophy in patients with mesial temporal sclerosis. Epilepsy Res. 219:107713. 10.1016/j.eplepsyres.2025.107713. 41314079

[ref119] Montaldi D, Mayes AR. 2010. The role of recollection and familiarity in the functional differentiation of the medial temporal lobes. Hippocampus. 20:1291–1314. 10.1002/hipo.20853. 20928828

[ref120] Morel A, Magnin M, Jeanmonod D. 1997. Multiarchitectonic and stereotactic atlas of the human thalamus. J Comp Neurol. 387:588–630. 10.1002/(SICI)1096-9861(19971103)387:4<588::AID-CNE8>3.0.CO;2-Z. 9373015

[ref121] Morgan VL, Rogers BP, Abou-Khalil B. 2015. Segmentation of the thalamus based on BOLD frequencies affected in temporal lobe epilepsy. Epilepsia. 56:1819–1827. 10.1111/epi.13186. 26360535 PMC4626388

[ref122] Moscovitch M, Cabeza R, Winocur G, Nadel L. 2016. Episodic memory and beyond: the hippocampus and neocortex in transformation. Annu Rev Psychol. 67:105–134. 10.1146/annurev-psych-113011-143733. 26726963 PMC5060006

[ref123] Mueller SG et al. 2010. Involvement of the thalamocortical network in TLE with and without mesiotemporal sclerosis. Epilepsia. 51:1436–1445. 10.1111/j.1528-1167.2009.02413.x. 20002143 PMC2888933

[ref124] Mullally SL, Intraub H, Maguire EA. 2012. Attenuated boundary extension produces a paradoxical memory advantage in amnesic patients. Curr Biol. 22:261–268. 10.1016/j.cub.2012.01.001. 22264610 PMC3315012

[ref125] Nestor PG et al. 2007. Episodic memory and neuroimaging of hippocampus and fornix in chronic schizophrenia. Psychiatry Res. 155:21–28. 10.1016/j.pscychresns.2006.12.020. 17395435

[ref126] Ohkawa T et al. 2013. Autoantibodies to epilepsy-related LGI1 in limbic encephalitis neutralize LGI1-ADAM22 interaction and reduce synaptic AMPA receptors. J Neurosci. 33:18161–18174. 10.1523/JNEUROSCI.3506-13.2013. 24227725 PMC3828467

[ref127] Park SA, Hahn JH, Kim JI, Na DL, Huh K. 2000. Memory deficits after bilateral anterior fornix infarction. Neurology. 54:1379–1382. 10.1212/WNL.54.6.1379. 10746616

[ref128] Park DC, Murman DL, Perry KD, Bruch LA. 2007. An autopsy case of limbic encephalitis with voltage-gated potassium channel antibodies. Eur J Neurol. 14:e5–e6. 10.1111/j.1468-1331.2007.01924.x. 17880556

[ref129] Parkin AJ, Rees JE, Hunkin NM, Rose PE. 1994. Impairment of memory following discrete thalamic infarction. Neuropsychologia. 32:39–51. 10.1016/0028-3932(94)90067-1. 8818153

[ref130] Parvizi J, Van Hoesen GW, Buckwalter J, Damasio A. 2006. Neural connections of the posteromedial cortex in the macaque. Proc Natl Acad Sci. 103:1563–1568. 10.1073/pnas.0507729103. 16432221 PMC1345704

[ref131] Patai EZ et al. 2015. Extent of hippocampal atrophy predicts degree of deficit in recall. Proc Natl Acad Sci USA. 112:12830–12833. 10.1073/pnas.1511904112. 26417089 PMC4611643

[ref132] Payne C, Cirilli L, Bachevalier J. 2017. An MRI study of the corpus callosum in monkeys: developmental trajectories and effects of neonatal hippocampal and amygdala lesions. Dev Psychobiol. 59:495–506. 10.1002/dev.21514. 28369850 PMC5421320

[ref133] Perry BAL, Mitchell AS. 2019. Considering the evidence for anterior and Laterodorsal thalamic nuclei as higher order relays to cortex. Front Mol Neurosci. 12:167. 10.3389/fnmol.2019.00167. PMC6616498 31333412

[ref134] Perry BAL, Lomi E, Mitchell AS. 2021. Thalamocortical interactions in cognition and disease: the mediodorsal and anterior thalamic nuclei. Neurosci Biobehav Rev. 130:162–177. 10.1016/j.neubiorev.2021.05.032. 34216651

[ref135] Philippi CL, Tranel D, Duff M, Rudrauf D. 2015. Damage to the default mode network disrupts autobiographical memory retrieval. Soc Cogn Affect Neurosci. 10:318–326. 10.1093/scan/nsu070. 24795444 PMC4350487

[ref136] Poreh A et al. 2006. Anterograde and retrograde amnesia in a person with bilateral fornix lesions following removal of a colloid cyst. Neuropsychologia. 44:2241–2248. 10.1016/j.neuropsychologia.2006.05.020. 16846621

[ref137] Preuss TM, Goldman-Rakic PS. 1987. Crossed corticothalamic and thalamocortical connections of macaque prefrontal cortex. J Comp Neurol. 257:269–281. 10.1002/cne.902570211. 3571529

[ref138] Qiao J et al. 2020. Functional and structural brain alterations in encephalitis with LGI1 antibodies. Front Neurosci. 14:304. 10.3389/fnins.2020.00304. PMC7146067 32317923

[ref139] Qiao J, Wang Z, Xin J, Wang S, Li A. 2025. Subcortical shape biomarkers reveal limbic and basal ganglia damage in anti-LGI1 encephalitis. Front Immunol. 16:1623577. 10.3389/fimmu.2025.1623577. 40688080 PMC12270846

[ref140] Raffelt DA et al. 2017. Investigating white matter fibre density and morphology using fixel-based analysis. NeuroImage. 144:58–73. 10.1016/j.neuroimage.2016.09.029. 27639350 PMC5182031

[ref141] Ramirez-Franco J et al. 2022. Patient-derived antibodies reveal the subcellular distribution and heterogeneous interactome of LGI1. Brain. 145:3843–3858. 10.1093/brain/awac218. 35727946

[ref142] Raschilas F et al. 2002. Outcome of and prognostic factors for herpes simplex encephalitis in adult patients: results of a Multicenter study. Clin Infect Dis. 35:254–260. 10.1086/341405. 12115090

[ref143] Rey A . 1959. Manuel du test de copie d’une figure complexe de a. Rey. Les Editions du Centre de Psychologie Appliquée, Paris, [published online ahead of print].

[ref144] Robertson RT, Kaitz SS. 1981. Thalamic connections with limbic cortex. I. Thalamocortical projections. J Comp Neurol. 195:501–525. 10.1002/cne.901950308. 7204659

[ref145] Romeo A et al. 2019. Early ictal recruitment of midline thalamus in mesial temporal lobe epilepsy. Ann Clin Transl Neurol. 6:1552–1558. 10.1002/acn3.50835. 31402630 PMC6689686

[ref146] Rosenberg DS et al. 2006. Involvement of medial Pulvinar thalamic nucleus in human temporal lobe seizures. Epilepsia. 47:98–107. 10.1111/j.1528-1167.2006.00375.x. 16417537

[ref147] Rosenberg DS, Mauguière F, Catenoix H, Faillenot I, Magnin M. 2009. Reciprocal Thalamocortical connectivity of the medial Pulvinar: a depth stimulation and evoked potential study in human brain. Cereb Cortex. 19:1462–1473. 10.1093/cercor/bhn185. 18936272

[ref148] Rudebeck SR et al. 2009. Fornix microstructure correlates with recollection but not familiarity memory. J Neurosci. 29:14987–14992. 10.1523/JNEUROSCI.4707-09.2009. 19940194 PMC2825810

[ref149] Rudebeck SR, Filippini N, Lee ACH. 2013. Can complex visual discrimination deficits in amnesia be attributed to the medial temporal lobe? An investigation into the effects of medial temporal lobe damage on brain connectivity. Hippocampus. 23:7–13. 10.1002/hipo.22056. 23233411 PMC3555392

[ref150] Saunders RC, Aggleton JP. 2007. Origin and topography of fibers contributing to the fornix in macaque monkeys. Hippocampus. 17:396–411. 10.1002/hipo.20276. 17372974

[ref151] Saunders RC, Mishkin M, Aggleton JP. 2005. Projections from the entorhinal cortex, perirhinal cortex, presubiculum, and parasubiculum to the medial thalamus in macaque monkeys: identifying different pathways using disconnection techniques. Exp Brain Res. 167:1–16. 10.1007/s00221-005-2361-3. 16143859

[ref152] Schulte U et al. 2006. The epilepsy-linked Lgi1 protein assembles into presynaptic Kv1 channels and inhibits inactivation by Kvβ1. Neuron. 49:697–706. 10.1016/j.neuron.2006.01.033. 16504945

[ref153] Scoville WB, Milner B. 1957. Loss of recentmemory after bilateral hippocampal lesions. J Neurol Neurosurg Psychiatry. 20:11–21. 10.1136/jnnp.20.1.11. 13406589 PMC497229

[ref154] Segobin S et al. 2019. Dissociating thalamic alterations in alcohol use disorder defines specificity of Korsakoff’s syndrome. Brain. 142:1458–1470. 10.1093/brain/awz056. 30879030

[ref155] Shibata H . 1993a. Efferent projections from the anterior thalamic nuclei to the cingulate cortex in the rat. J Comp Neurol. 330:533–542. 10.1002/cne.903300409. 8320343

[ref156] Shibata H . 1993b. Direct projections from the anterior thalamic nuclei to the retrohippocampal region in the rat. J Comp Neurol. 337:431–445. 10.1002/cne.903370307. 7506716

[ref157] Shibata H, Yukie M. 2003. Differential thalamic connections of the posteroventral and dorsal posterior cingulate gyrus in the monkey. Eur J Neurosci. 18:1615–1626. 10.1046/j.1460-9568.2003.02868.x. 14511340

[ref158] Shimosaka S, So YT, Simon RP. 1992. Distribution of HSP72 induction and neuronal death following limbic seizures. Neurosci Lett. 138:202–206. 10.1016/0304-3940(92)90915-T. 1376869

[ref159] Silva J, Wang G, Cowell JK. 2011. The temporal and spatial expression pattern of the LGI1 epilepsy predisposition gene during mouse embryonic cranial development. BMC Neurosci. 12:43. 10.1186/1471-2202-12-43. 21569517 PMC3120723

[ref160] Smith SM, Nichols TE. 2009. Threshold-free cluster enhancement: addressing problems of smoothing, threshold dependence and localisation in cluster inference. NeuroImage. 44:83–98. 10.1016/j.neuroimage.2008.03.061. 18501637

[ref161] van Sonderen A et al. 2016. Anti-LGI1 encephalitis. Neurology. 87:1449–1456. 10.1212/WNL.0000000000003173. 27590293

[ref162] van Sonderen A, Petit-Pedrol M, Dalmau J, Titulaer MJ. 2017. The value of LGI1, Caspr2 and voltage-gated potassium channel antibodies in encephalitis. Nat Rev Neurol. 13:290–301. 10.1038/nrneurol.2017.43. 28418022

[ref163] Soskic S et al. 2025. Thalamus involvement in genetic frontotemporal dementia assessed using structural and diffusion MRI: a GENFI study. Brain Commun. 7:fcaf420. 10.1093/braincomms/fcaf420. PMC12612583 41245435

[ref164] Soulier H et al. 2023. The anterior and pulvinar thalamic nuclei interactions in mesial temporal lobe seizure networks. Clin Neurophysiol. 150:176–183. 10.1016/j.clinph.2023.03.016. 37075682

[ref165] Spanò G et al. 2020a. Dreaming with hippocampal damage Colgin LL, Irish M, Payne J, editors. elife. 9:e56211. 10.7554/eLife.56211. 32508305 PMC7279885

[ref166] Spanò G et al. 2020b. Sleeping with hippocampal damage. Curr Biol. 30:523–529.e3. 10.1016/j.cub.2019.11.072. 31956024 PMC6997880

[ref167] Squire LR . 2017. Memory for relations in the short term and the long term after medial temporal lobe damage. Hippocampus. 27:608–612. 10.1002/hipo.22716. 28188665 PMC5889104

[ref168] Squire LR, Bayley PJ. 2007. The neuroscience of remote memory. Curr Opin Neurobiol. 17:185–196. 10.1016/J.CONB.2007.02.006. 17336513 PMC2277361

[ref169] Sweeney-Reed CM et al. 2021. The role of the anterior nuclei of the thalamus in human memory processing. Neurosci Biobehav Rev. 126:146–158. 10.1016/j.neubiorev.2021.02.046. 33737103

[ref170] Tomás Pereira I, Agster KL, Burwell RD. 2016. Subcortical connections of the perirhinal, postrhinal, and entorhinal cortices of the rat. I afferents Hippocampus. 26:1189–1212. 10.1002/hipo.22603. 27119220 PMC5070464

[ref171] Tournier J-D et al. 2019. *MRtrix3*: a fast, flexible and open software framework for medical image processing and visualisation. NeuroImage. 202:116137. 10.1016/j.neuroimage.2019.116137. 31473352

[ref172] Tregidgo HFJ et al. 2023. Domain-agnostic segmentation of thalamic nuclei from joint structural and diffusion MRI. In: Greenspan H, Madabhushi A, Mousavi P, Salcudean S, Duncan J, Syeda-Mahmood T, Taylor R, editors. Medical image computing and computer assisted intervention – MICCAI 2023. MICCAI 2023. Lecture notes in computer science, Vol. 14227. Springer, pp 247–257.

[ref173] Tröscher AR et al. 2017. Selective limbic blood–brain barrier breakdown in a feline model of limbic encephalitis with LGI1 antibodies. Front Immunol. 8:1364. 10.3389/fimmu.2017.01364. 29093718 PMC5651237

[ref174] Tsivilis D et al. 2008. A disproportionate role for the fornix and mammillary bodies in recall versus recognition memory. Nat Neurosci. 11:834–842. 10.1038/nn.2149. 18552840

[ref175] Tung H, Pan SY, Lan TH, Lin YY, Peng SJ. 2022. Characterization of hippocampal-thalamic-cortical morphometric reorganization in temporal lobe epilepsy. Front Neurol. 12:810186. 10.3389/fneur.2021.810186. 35222230 PMC8866816

[ref176] Turski L et al. 1986. Seizures produced by pilocarpine: neuropathological sequelae and activity of glutamate decarboxylase in the rat forebrain. Brain Res. 398:37–48. 10.1016/0006-8993(86)91247-3. 3801899

[ref177] Urgolites ZJ, Hopkins RO, Squire LR. 2017. Medial temporal lobe and topographical memory. Proc Natl Acad Sci USA. 114:8626–8630. 10.1073/pnas.1708963114. 28739918 PMC5559052

[ref178] Van Groen T, Wyss JM. 1992. Projections from the laterodorsal nucleus of the thalamus to the limbic and visual cortices in the rat. J Comp Neurol. 324:427–448. 10.1002/cne.903240310. 1383292

[ref58] van Groen T, Kadish I, Wyss JM. 2002. The role of the laterodorsal nucleus of the thalamus in spatial learning and memory in the rat. Behav Brain Res. 136:329–337. 10.1016/s0166-4328(02)00199-7. 12429394

[ref179] Vann SD et al. 2009. Impaired recollection but spared familiarity in patients with extended hippocampal system damage revealed by 3 convergent methods. Proc Natl Acad Sci USA. 106:5442–5447. 10.1073/pnas.0812097106. 19289844 PMC2664061

[ref180] Varela C, Kumar S, Yang JY, Wilson MA. 2014. Anatomical substrates for direct interactions between hippocampus, medial prefrontal cortex, and the thalamic nucleus reuniens. Brain Struct Funct. 219:911–929. 10.1007/s00429-013-0543-5. 23571778 PMC4179252

[ref181] Vertes RP . 2006. Interactions among the medial prefrontal cortex, hippocampus and midline thalamus in emotional and cognitive processing in the rat. Neuroscience. 142:1–20. 10.1016/j.neuroscience.2006.06.027. 16887277

[ref182] Vertes RP, Hoover WB, Szigeti-Buck K, Leranth C. 2007. Nucleus reuniens of the midline thalamus: link between the medial prefrontal cortex and the hippocampus. Brain Res Bull. 71:601–609. 10.1016/j.brainresbull.2006.12.002. 17292803 PMC4997812

[ref183] Vidal JP et al. 2024. Robust thalamic nuclei segmentation from T1-weighted MRI using polynomial intensity transformation. Brain Struct Funct. 229:1087–1101. 10.1007/s00429-024-02777-5. 38546872 PMC11147736

[ref184] Voets NL et al. 2015. Thalamo-cortical disruption contributes to short-term memory deficits in patients with medial temporal lobe damage. Cereb Cortex. 25:4584–4595. 10.1093/cercor/bhv109. 26009613 PMC4816801

[ref185] Wagner J, Weber B, Elger CE. 2015. Early and chronic gray matter volume changes in limbic encephalitis revealed by voxel-based morphometry. Epilepsia. 56:754–761. 10.1111/epi.12968. 25809952

[ref186] Warburton EC, Baird AL, Aggleton JP. 1997. Assessing the magnitude of the allocentric spatial deficit associated with complete loss of the anterior thalamic nuclei in rats. Behav Brain Res. 87:223–232. 10.1016/S0166-4328(97)02285-7. 9331491

[ref187] Warrington E . 1984. The recognition memory test. NFER-Nelson.

[ref188] Warrington EK . 1996. The Camden memory tests. Psychology Press.

[ref189] Warrington EK, Weiskrantz L. 1982. Amnesia: a disconnection syndrome? Neuropsychologia. 20:233–248. 10.1016/0028-3932(82)90099-9. 7121792

[ref190] Wasserthal J, Neher P, Maier-Hein KH. 2018. TractSeg - fast and accurate white matter tract segmentation. NeuroImage. 183:239–253. 10.1016/j.neuroimage.2018.07.070. 30086412

[ref191] Webster MJ, Bachevalier J, Ungerleider LG. 1993. Subcortical connections of inferior temporal areas TE and TEO in macaque monkeys. J Comp Neurol. 335:73–91. 10.1002/cne.903350106. 8408774

[ref192] Wechsler D . 1997. Wechsler memory scale-III: Wechsler memory scale administartion and scoring manual. The Psychological Corporation.

[ref193] Winocur G . 1985. The hippocampus and thalamus: their roles in short- and long-term memory and the effects of interference. Behav Brain Res. 16:135–152. 10.1016/0166-4328(85)90088-9. 4041213

[ref194] Wisse LEM et al. 2021. Hippocampal subfield volumetry from structural isotropic 1 mm3 MRI scans: a note of caution. Hum Brain Mapp. 42:539–550. 10.1002/hbm.25234. 33058385 PMC7775994

[ref195] Wixted JT, Squire LR. 2004. Recall and recognition are equally impaired in patients with selective hippocampal damage. Cogn Affect Behav Neurosci. 4:58–66. 10.3758/CABN.4.1.58. 15259889

[ref196] Wixted JT, Squire LR. 2011. The familiarity/recollection distinction does not illuminate medial temporal lobe function: response to Montaldi and Mayes. Trends Cogn Sci. 15:340–341. 10.1016/j.tics.2011.06.006. 21763175 PMC3457644

[ref197] Wolff M, Halassa MM. 2024. The mediodorsal thalamus in executive control. Neuron. 112:893–908. 10.1016/j.neuron.2024.01.002. 38295791

[ref198] Yanagihara M, Ono K, Niimi K. 1985. Thalamic projections to the hippocampal formation in the cat. Neurosci Lett. 61:31–35. 10.1016/0304-3940(85)90396-9. 4080258

[ref199] Yendiki A et al. 2011. Automated probabilistic reconstruction of white-matter pathways in health and disease using an atlas of the underlying anatomy. Front Neuroinform. 5:23. 10.3389/fninf.2011.00023. PMC3193073 22016733

[ref200] Yeterian EH, Pandya DN. 1985. Corticothalamic connections of the posterior parietal cortex in the rhesus monkey. J Comp Neurol. 237:408–426. 10.1002/cne.902370309. 4044894

[ref201] Yonelinas A, Hawkins C, Abovian A, Aly M. 2024. The role of recollection, familiarity, and the hippocampus in episodic and working memory. Neuropsychologia. 193:108777. 10.1016/j.neuropsychologia.2023.108777. 38141964 PMC10872349

[ref202] Zamboni G et al. 2013. Resting functional connectivity reveals residual functional activity in Alzheimer’s disease. Biol Psychiatry. 74:375–383. 10.1016/J.BIOPSYCH.2013.04.015. 23726515

[ref203] Zarkali A et al. 2020. Fiber-specific white matter reductions in Parkinson hallucinations and visual dysfunction. Neurology. 94:e1525. 10.1212/WNL.0000000000009014. 32094242 PMC7251523

[ref204] Zheng L et al. 2018. Meta-analysis of voxel-based morphometry studies of gray matter abnormalities in patients with mesial temporal lobe epilepsy and unilateral hippocampal sclerosis. Brain Imaging Behav. 12:1497–1503. 10.1007/s11682-017-9797-5. 29302917

[ref205] Zhou Y-D et al. 2012. Epilepsy gene LGI1 regulates postnatal developmental Remodeling of Retinogeniculate synapses. J Neurosci. 32:903–910. 10.1523/JNEUROSCI.5191-11.2012. 22262888 PMC3342858

[ref206] Zola-Morgan S, Squire LR, Amaral DG. 1989. Lesions of the hippocampal formation but not lesions of the fornix or the mammillary nuclei produce long-lasting memory impairment in monkeys. J Neurosci. 9:898–913. 10.1523/JNEUROSCI.09-03-00898.1989. 2494309 PMC6569954

